# sTRAIL-iRGD is a promising therapeutic agent for gastric cancer treatment

**DOI:** 10.1038/s41598-017-00688-6

**Published:** 2017-04-03

**Authors:** Ying Huang, Xihan Li, Huizi Sha, Lianru Zhang, Xinyu Bian, Xiao Han, Baorui Liu

**Affiliations:** 1 Department of Pain of Drum Tower Hospital, Medical School of Nanjing University, Nanjing, Jiangsu China; 2 Central Laboratory, Nanjing Integrated Traditional Chinese and Western Medicine Hospital, Affiliated Nanjing University of Chinese Medicine, Nanjing, China; 3 The Comprehensive Cancer Centre of Drum Tower Hospital, Medical School of Nanjing University & Clinical Cancer Institute of Nanjing University, Nanjing, Jiangsu China; 4 Key Laboratory of Human Functional Genomics of Jiangsu Province, Jiangsu Diabetes Center, Nanjing Medical University, Nanjing, Jiangsu China

## Abstract

Tumor necrosis factor-related apoptosis-inducing ligand (TRAIL) selectively kills tumor cells and augments chemotherapeutics *in vivo*. Here, we developed sTRAIL-iRGD, a recombinant protein consisting of sTRAIL fused to CRGDKGPDC, a C-terminal end binding peptide with an integrin-binding arginine-glycine-aspartic acid (iRGD) motif. CRGDKGPDC is a tumor-homing peptide with high penetration into tumor tissue and cells. We found that sTRAIL-iRGD internalized into cultured gastric cancer tumor cells and localized to both the tumor mass *in vivo* and three-dimensional multicellular spheroids *in vitro*. sTRAIL-iRGD had an antitumor effect in tumor cell lines, multicellular spheroids and nude mice with tumors. Repeated treatment with sTRAIL-iRGD reduced tumor growth and volume *in vivo*. Mice treated with sTRAIL-iRGD and paclitaxel (PTX) in combination showed no sign of sTRAIL-iRGD-related liver toxicity. Our data suggest that sTRAIL-iRGD is a promising anti-gastric cancer agent with high selectivity and limited systemic toxicity.

## Introduction

Despite recent progress in cancer immune therapy, radiotherapy and chemotherapy, gastric cancer continues to have high mortality rates globally, especially in the Eastern world^1–3^. Programmed cell death is a biological defense mechanism whereby defective and harmful cells are eliminated^4^. Disruption of programmed cell death signaling pathways may result in elevated cell proliferation and eventually cancer. Tumor necrosis factor (TNF)-related apoptosis-inducing ligand (TRAIL), a homotrimeric type II transmembrane protein, plays a role in apoptosis induction^5^. sTRAIL, a soluble form of TRAIL, can be proteolytically cleaved, and can maintain its original tumor cell killing activity^6, 7^. sTRAIL consists of 168 carboxy-terminal amino acids from the TRAIL extracellular domain (19.6 kD)^8^. Most human primary cells exhibited no obvious cytotoxicity as a result of soluble TRAIL variants, which were also tolerated by chimpanzees and mice^9^. A few recombinant sTRAIL derivatives exhibit antitumor activities *in vitro* and in mouse models bearing tumors. sTRAIL is a new and original therapeutic approach, which can induce apoptosis in diverse tumors^8^. However, TRAIL is limited for therapeutic purposes due to cytotoxicity in hepatocytes^10, 11^. In addition, circulating sTRAIL protein exhibits a short half-life, such that its bioavailability at the tumor site may be inadequate^12^.

New TRAIL-based fusion proteins are needed to overcome the current limitations of this potentially efficacious therapeutic strategy. In the present paper, we investigated the development of sTRAIL complexes equipped with targeting ligands directed to the tumor vasculature. Tissue specificity and enhanced preservation of sTRAIL complexes within the tumor vasculature will improve bioavailability in tumor tissues and potentially mitigate off-target toxicity.

Following the establishment of the multicellular spheroids (MCS) model by Sutherland, *et. al*.^13, 14^ in the 1970s, three-dimensional (3D) MCS has been developed as a simple and practical *in vitro* model reflecting natural solid tumor properties. 3D MCS culture conditions generate an extracellular matrix (ECM) that obstructs drug penetration into tumor tissues^15, 16^. Large MCS (>200 μm in diameter) may be composed of three regions, including an actively growing peripheral cell population, an intermediate resting zone and a necrotic core^17, 18^. Minchinton, *et al*.^19^ suggest that MCS may be ideal for researching drug penetration *in vivo* into a multilayer cell culture system. We undertook this study using 3D MCS with gastric cancer cells to elucidate sTRAIL antitumor activities and explore sTRAIL as an antitumor drug.

To circumvent the issue of low drug penetration into solid tumors and to efficiently deliver sTRAIL into the tumor, we fused CRGDKGPDC, a C-terminal end binding peptide with an integrin-binding arginine-glycine-aspartic acid (iRGD) motif, to sTRAIL. Tumor-homing is regulated by the iRGD motif via selective αvβ3/5 integrin binding on the surfaces of diverse tumor cell types and tumor vessel endothelium^20^. CRGDKGPDC is proteolytically cleaved to CRGDK/R, which binds and activates the receptor Neuropilin-1 (NRP-1), as a transmembrane protein, involves in mediating the peptide’s penetration into tissues^21^. Hence, the addition of the iRGD motif enhances sTRAIL-iRGD tumor-specific tissue penetration. We also examined sTRAIL-iRGD anticancer activity in combination with paclitaxel (PTX) *in vitro*, using the 3D MCS model, and *in vivo*.

## Results

### Construction, production and characterization of recombinant fusion proteins

**Figure 1:**
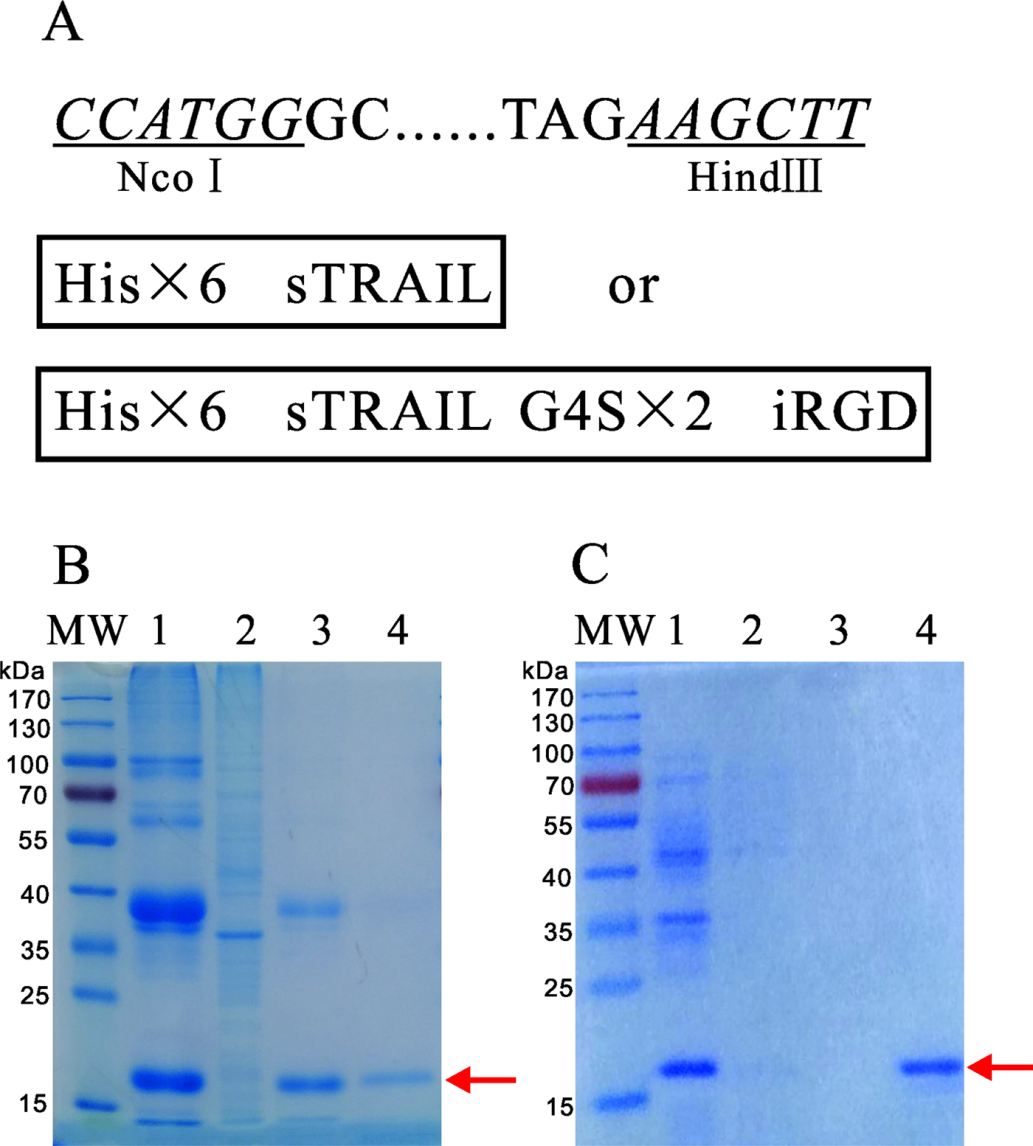
Construction of sTRAIL-iRGD. Restriction sites are underlined; His × 6 and G4S tags are shown in boxes. (**A**) sTRAIL purified via nickel-affinity chromatography (red arrows in the upper panel). (**B**) Lanes 1–3: sTRAIL at 10%, 30% and 50% eluent times; Lane 4: sTRAIL fusion protein expression was validated by SDS-PAGE (red arrows) at 100% eluent times. sTRAIL purified via nickel-affinity chromatography (red arrows in the upper panel) (**C**). Lanes 1–3: sTRAIL-iRGD at 10%, 30% and 50% eluent times; Lane 4: sTRAIL-iRGD fusion protein expression was validated by SDS-PAGE (red arrows) at 100% eluent times.

sTRAIL and sTRAIL-iRGD genes in frame were cloned separately into NcoI and HindIII restriction sites of the pET28a plasmid to add His tags (Fig. 1A). After IPTG induction, three protein bands appeared at 20 kDa (sTRAIL) and 22 kDa (sTRAIL-iRGD) in coomassie blue-stained SDS-PAGE. These results validated the successful production of soluble sTRAIL and sTRAIL-iRGD. A majority of the recombinant proteins were successfully purified (Fig. 1B,C).

### Recombinant protein internalization into cells

**Figure 2:**
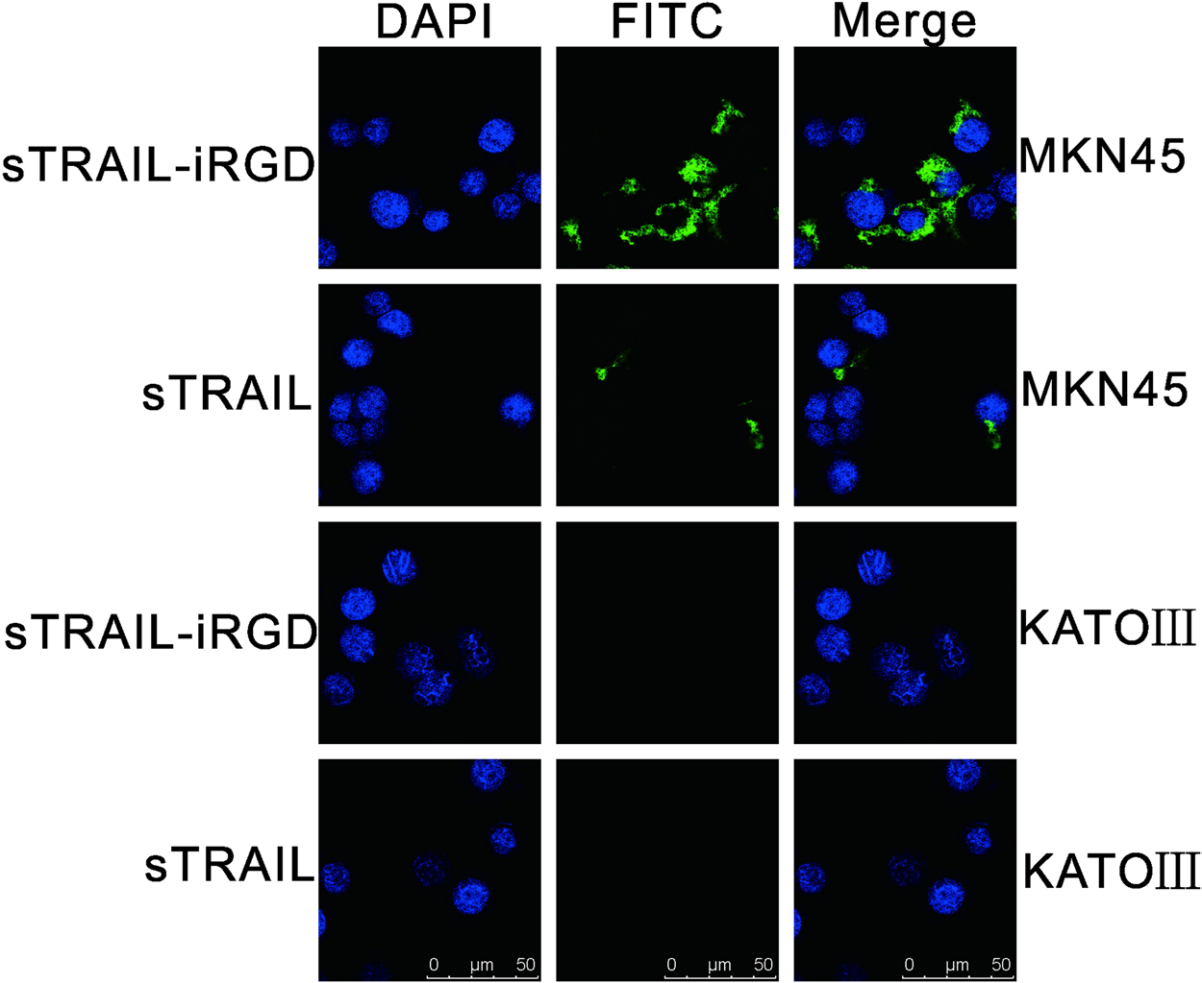
sTRAIL-iRGD and sTRAIL internalization into MKN45 and KATOIII cells. MKN45 cells, which are positive for NRP-1 and DR4, and NRP-1-deficient, DR4-deficient KATOIII cells were incubated with FITC-labeled fusion proteins. Nuclei were stained with DAPI (blue). Proteins were visualized by confocal microscopy.

Internalization of the recombinant proteins by human gastric carcinoma cells MKN45 and KATOIII was assessed by LSCM using FITC-labeled sTRAIL-iRGD and sTRAIL. sTRAIL bound to MKN45 cells (Fig. 2), which expresses high levels of NRP-1 and designated death receptor–4 (DR4), which is the receptor for the proapoptotic ligand TRAIL and contains a cytoplasmic “death domain” capable of engaging the cell suicide apparatus and the apoptotic proteases that compose it. sTRAIL also exhibited negligible binding to KATOIII cells, which expresses low levels of NRP-1 and DR4. In contrast, sTRAIL-iRGD more effectively bound to and was taken up into MKN45 cells, but did not bind to or internalize into NRP-1-deficient KATOIII cells. sTRAIL-iRGD internalization was rapid, and was detectable in MKN45 cells after 30 min incubation.

### Reduction of cell viability by recombinant proteins

**Figure 3:**
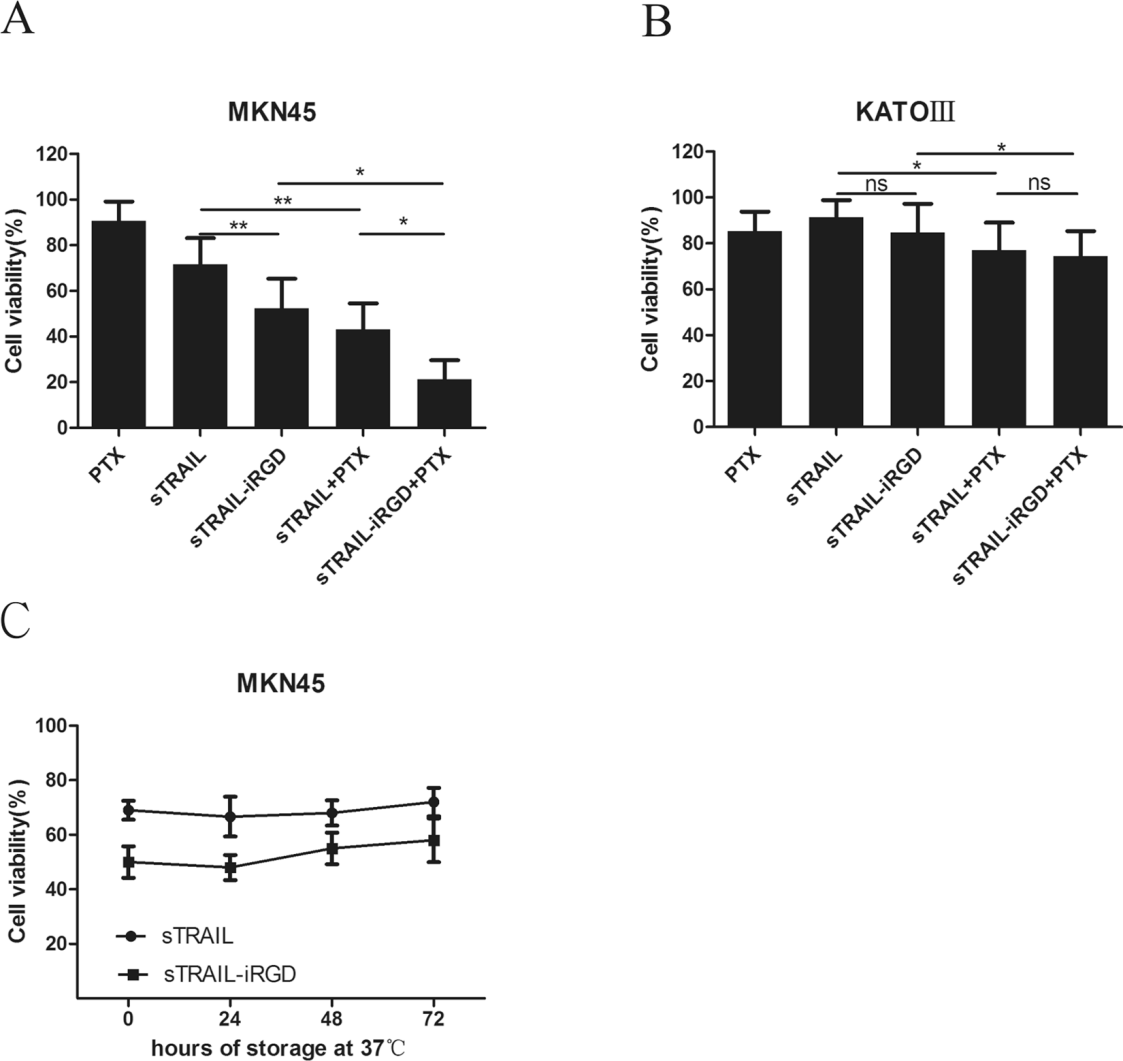
sTRAIL-iRGD + PTX cytotoxicity in tumor cell lines. sTRAIL-iRGD or sTRAIL combined with PTX were added to MKN45 (**A**) and KATOIII (**B,C**), comparison of the stability of sTRAIL-iRGD. MKN45 cells were treated with sTRAIL-iRGD and sTRAIL that was stored for either 24, 48 or 72 h prior treatment at 37 °C. Cell suspensions and incubated for 24 h. Cell viability was measured by MTT assay. All data are expressed as means ± standard deviation (SD), n = 3, and represent the means of three independent experiments. Statistical analyses were performed using the Student t-test and one-way ANOVA. *p < 0.05; **p < 0.01; ns, not significant.

To confirm sTRAIL-iRGD pharmacological activity *in vitro*, MKM45 and KATOIII cell viability was assessed via MTT assay. The commonly used anti-cancer drug, PTX, targets microtubules in a wide variety of human malignancies, including lung, breast and gastric carcinomas. PTX can induce cell cycle arrest in G2-M phase and induce cancer cell apoptosis by disrupting normal microtubule breakdown during cell division. sTRAIL-iRGD or sTRAIL in combination with PTX exhibited antiproliferative activity in MKM45 cells at 100 ng/mL (Fig. 3A), although sTRAIL-iRGD inhibited cell growth more strongly than sTRAIL. We found that sTRAIL-iRGD or sTRAIL combined with PTX (4 μg/mL) decreased cell viability at 24 h as compared to any of the therapeutics alone. For KATOIII cells (Fig. 3B), sTRAIL-iRGD and sTRAIL exhibited similar (not significantly different) anti-proliferative activity, although sTRAIL-iRGD inhibited proliferating KATOIII slightly more than sTRAIL. sTRAIL-iRGD and sTRAIL combined with PTX (4 μg/mL) resulted in a slightly decreased cell viability as compared to any single therapeutic alone. We compared the stability of sTRAIL-iRGD and sTRAIL by storing them for up to 72 h at 37 °C. This revealed that sTRAIL-iRGD and sTRAIL retained the pro-apoptotic activity towards MKN45 cells from all storage time points evaluated (Fig. 3C).

**Figure 4:**
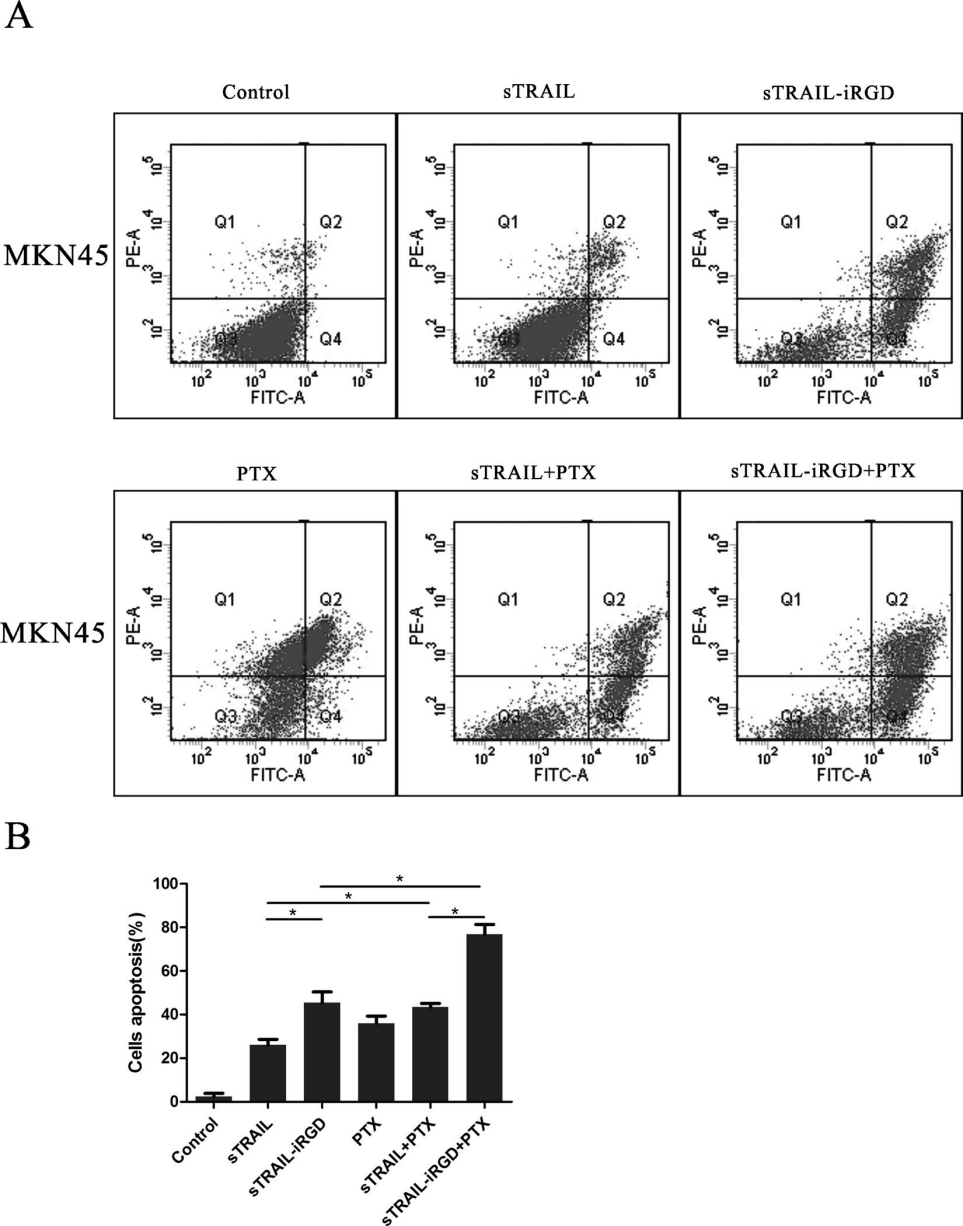
sTRAIL-iRGD + PTX induced tumor cell apoptosis *in vitro*. Cell apoptosis rates in MKN45 after treatment (monotherapy or recombinant proteins combined with PTX) as measured by Annexin V/PI apoptosis assay. (**A**) Percentage of apoptotic cells detected by flow cytometry. (**B**) The average of number of apoptotic cells (±SD) was shown. *p < 0.05; ns, not significant.

MKN45 cell apoptosis rates were analyzed by flow cytometry using the Annexin-V-FLUOS Staining apoptosis assay. Differential staining results are shown as percentages of positive cells compared to the total number of cells. Q4 shows early apoptosis cells, Q2 shows late apoptosis cells, and Q2 plus Q4 indicates gastric cells of apoptosis (Fig. 4A). Our results revealed that sTRAIL induced low apoptotic rates in the highly metastatic MKN45 cells (ratio of early and late apoptosis, 26.0 + 4.5%) (Fig. 4A). By comparison, sTRAIL-iRGD induced both apoptosis and necrosis (ratio of early and late apoptosis, 45.4 ± 8.5%) (Fig. 4A). According to these findings, sTRAIL-iRGD increases apoptosis *in vitro* and is effectively internalized into cancer cells (Fig. 4B). Apoptosis was increased with PTX in combination with sTRAIL-iRGD (ratio of early and late apoptosis, 76.8 ± 7.9%).

### MCS growth inhibition and recombinant protein internalization

**Figure 5:**
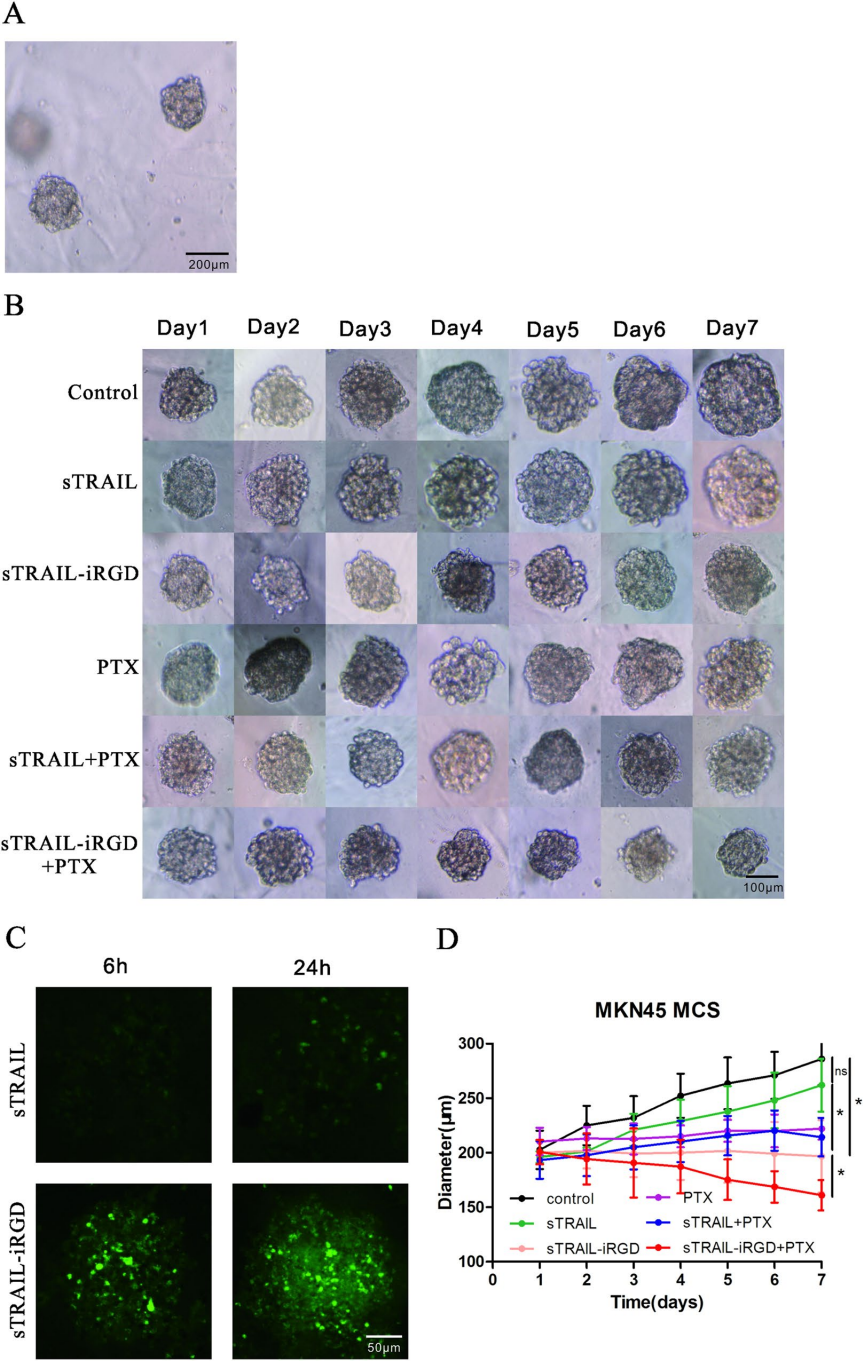
Visualization of MKN45 3D MCS. MKN45 3D MCS captured by optical microscopy (scale bar = 200 μm). (**A**) Representative images of MCS treated with culture medium, sTRAIL, sTRAIL-iRGD, PTX, sTRAIL + PTX or sTRAIL-iRGD + PTX. (**B**) Control MCS were cultured in RPMI1640 medium alone (scale bar = 100 μm). LSCM images of MKN45 MCS incubated with FITC-labeled sTRAIL or sTRAIL-iRGD for 6 and 24 h (scale bar = 50 μm). (**C**) Growth inhibition assay in MKN45 MCS. (**D**) MCS growth curves after treatment. All data are expressed as means ± standard deviation (SD), n = 3. *p < 0.05.

MKN45 3D MCS were symmetrically and spherically shaped with an average diameter of about 200 μm after seven days study in culture (Fig. 5A). MKN45 MCS were exposed to medium containing a recombinant protein alone (100 ng/mL), PTX alone (4 μg/mL) or PTX (4 μg/mL) plus protein (100 ng/mL) for 24 h. MCS were then incubated for six days with daily media replacement. The MCS grew during the seven days (Fig. 5B), and cells became very compact. Mean diameter changes for all six groups are presented in Fig. 5D. Diameters in the control and sTRAIL groups expanded from 203 to 286 μm and 196 to 262 μm, respectively. MKN45 MCS treated with sTRAIL-iRGD ceased growing and even became smaller, with a mean diameter reduction of 200 to 197 μm. On day seven, average diameter differences between sTRAIL and sTRAIL-iRGD-treated cells became significant (p < 0.05). sTRAIL-iRGD combined with PTX caused a greater MCS diameter reduction than did sTRAIL-iRGD alone (p < 0.05). These results show that sTRAIL-iRGD combined with PTX inhibits MCS growth more efficiently than any of the therapeutics alone. sTRAIL-iRGD internalization into MCS was also analyzed. At 6 and 24 h, sTRAIL-iRGD-associated fluorescence was stronger than that of sTRAIL (Fig. 5C).

### sTRAIL-iRGD penetration into tumor tissues

**Figure 6:**
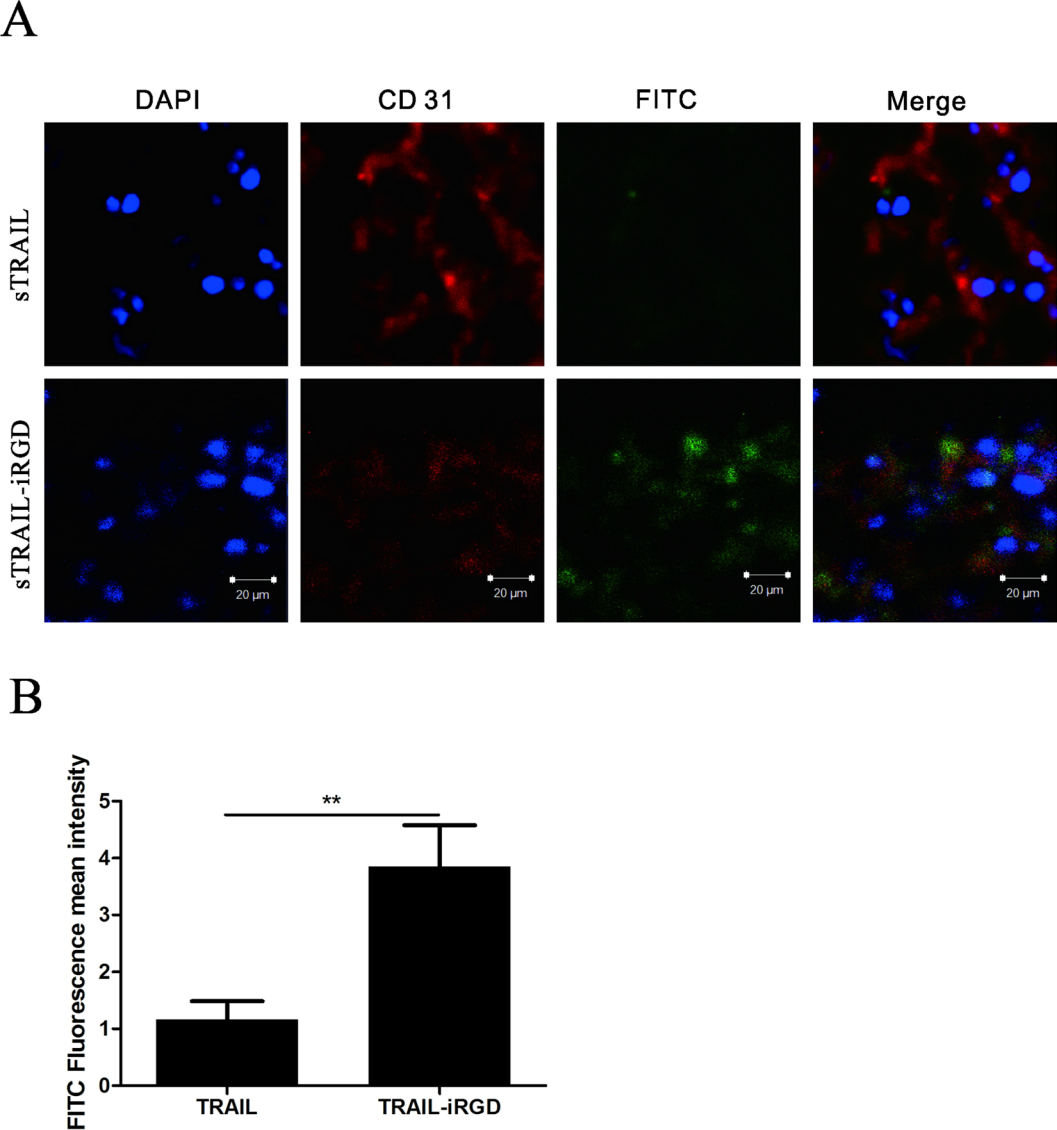
sTRAIL and sTRAIL-iRGD protein tumor penetration in MKN45 cell-injected mice 1 h post-injection. Frozen sections were examined under a confocal microscope. (**A**) Blood vessels were visualized with anti-CD31 (red), nuclei were stained with DAPI (blue), and green represents sTRAIL or sTRAIL-iRGD. Bars represent 20 μm. Quantification of fluorescence intensity. (**B**) Error bars denote SD. **p ≤ 0.01.

We assessed recombinant protein penetration into tumor tissues derived from MKN45 tumor-bearing mice using FITC-labeled sTRAIL and sTRAIL-iRGD (Fig. 6A). An anti-CD31 antibody was visualized bound to blood vessels using a Cy3-conjugated secondary antibody (red). sTRAIL was localized in the vicinity of blood vessels 1 h post-injection. In contrast, the sTRAIL-iRGD peptide was seen to penetrate extravascular tumor tissue, with strong sTRAIL-iRGD signal detectable in all sections from an entire tumor. sTRAIL-iRGD could be found on a greater field in the tumor tissues in comparison with sTRAIL. When we compared FITC fluorescence mean intensities, sTRAIL signaling was four times weaker than that of sTRAIL-iRGD (Fig. 6B).

### Inhibitory effects of sTRAIL-iRGD in a xenograft model

**Figure 7:**
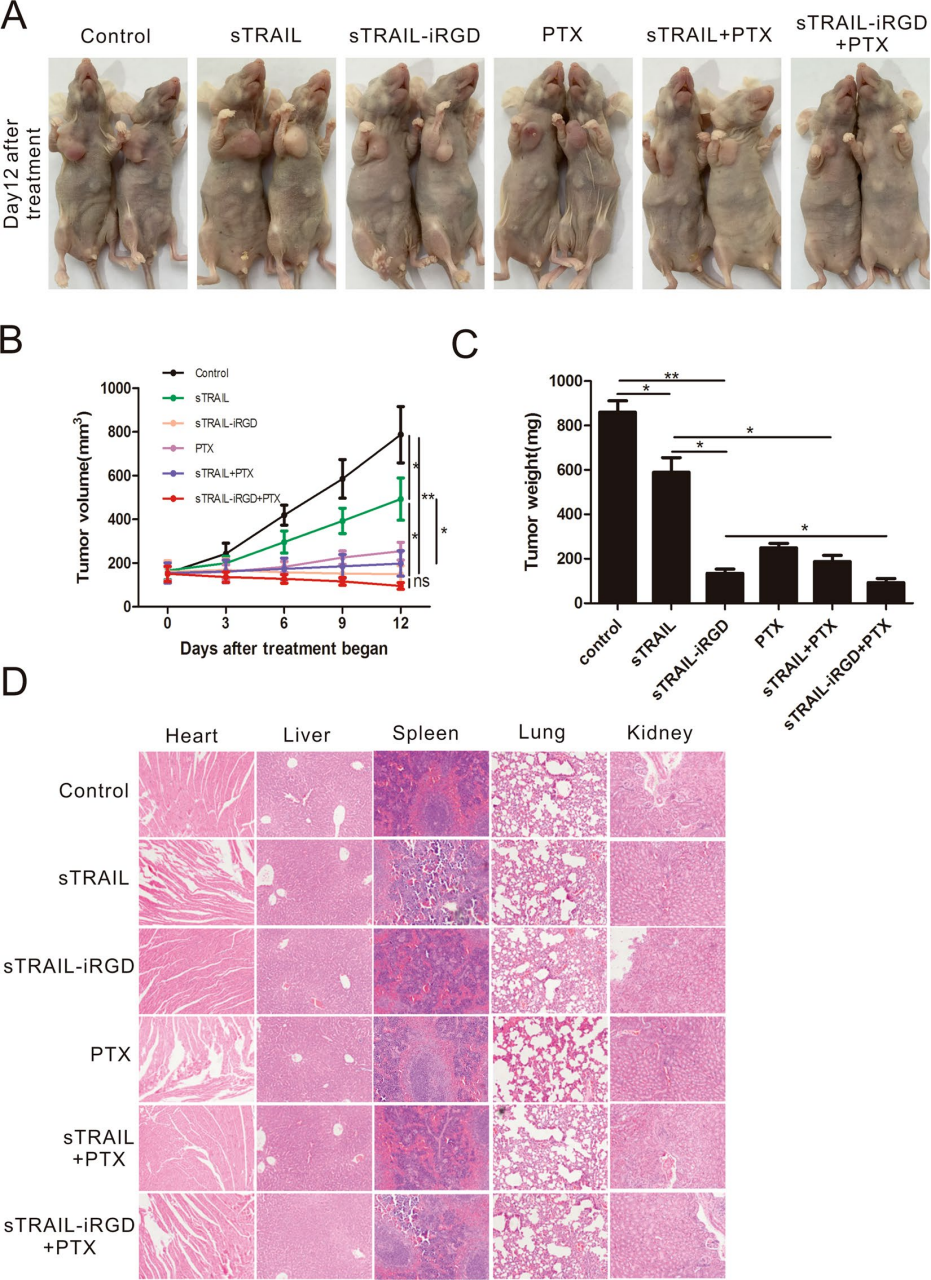
sTRAIL-iRGD suppresses tumor growth with no toxic effects in a xenograft model. MKN45 cells were subcutaneously injected into the right armpits of nude mice and allowed to establish xenografts for two weeks. (**A**) PBS, sTRAIL, sTRAIL-iRGD, PTX, sTRAIL + PTX or sTRAIL-iRGD + PTX were intraperitoneally injected into mice. Photo taken at 12 days post treatment. MKN45 xenograft volumes from treated animals (n = 6). (**B**) Mean tumor weights after treatment. (**C**) Morphological details were investigated using H&E staining. (**D**) Scale bar represents 50 μm. *p ≤ 0.05; **p ≤ 0.01; ns, not significant.

To examine sTRAIL-iRGD antitumor effects *in vivo*, we systemically administered the peptide into nude mice with established MKN45 tumors. We injected sTRAIL, sTRAIL-iRGD, PTX, sTRAIL + PTX or sTRAIL-iRGD + PTX intraperitoneally at 10 mg/kg of mouse body weight every three days. MKN45 tumor sizes increased quickly in mice injected with either PBS (control) or sTRAIL. In comparison, inhibition of tumor growth was evident as early as three days after treatment in sTRAIL-iRGD, PTX, sTRAIL + PTX or sTRAIL-iRGD + PTX treated mice. A representative image of a nude mouse 15 days post-treatment is presented in Fig. 7A. Time-dependent changes in MKN45 tumor growth are shown in Fig. 7B. Our results showed that sTRAIL-iRGD treatment effectively suppressed MKN45 tumor growth in the xenograft model, and this ression was enhanced by the addition of PTX (Fig. 7C).

To assess the potential toxicity of sTRAIL-iRGD in mice, H&E-stained kidney, lung, heart, liver and spleen sections were examined after peptide treatment. There were no obvious changes in these organs following treatment with sTRAIL-iRGD or sTRAIL-iRGD + PTX as compared to controls (Fig. 7D). Additionally, no skin abnormalities around the injection sites were observed.

**Figure 8:**
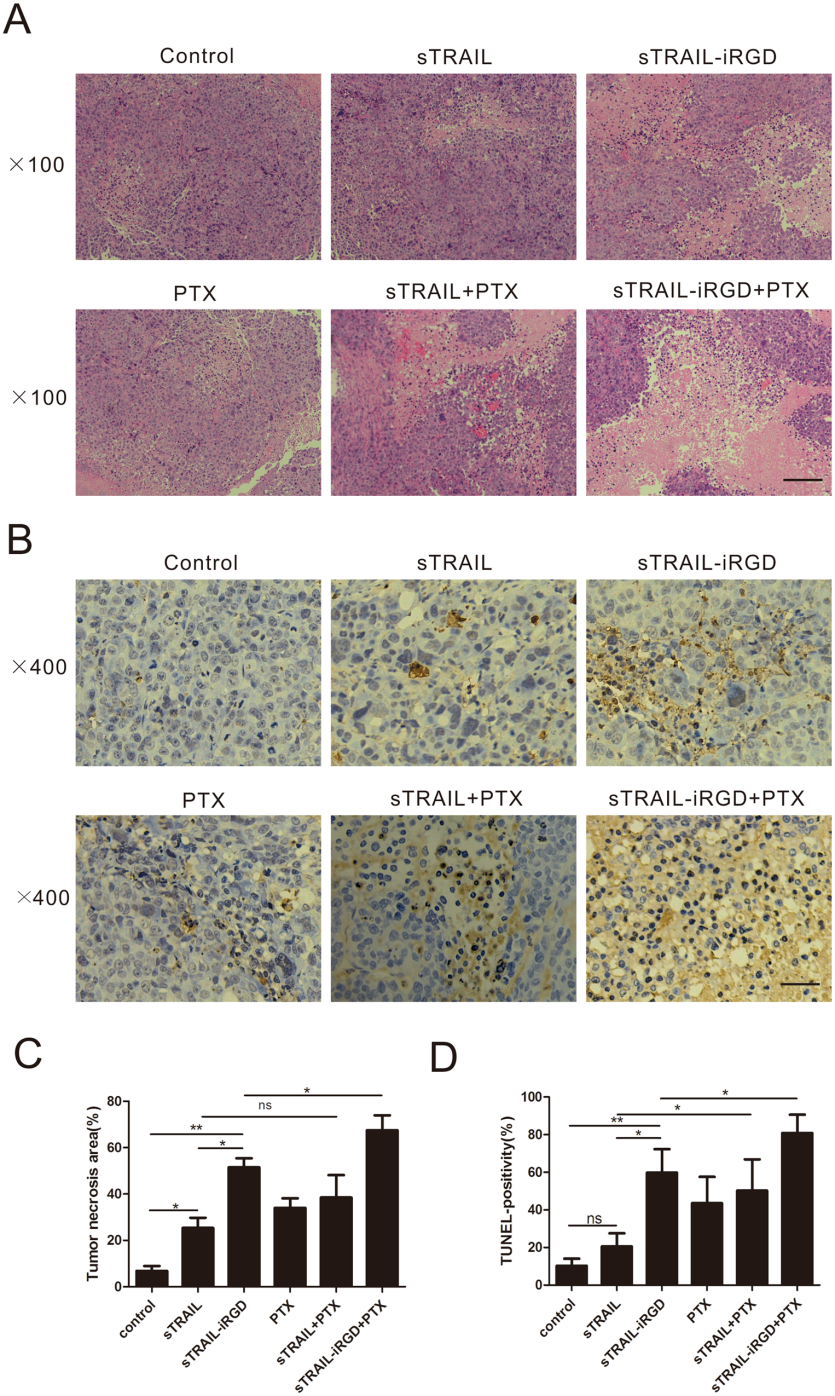
Cell death analysis in sTRAIL-iRGD-treated MKN45 tumors. H&E staining of tumor sections from mice intraperitoneally injected with PBS, sTRAIL, sTRAIL-iRGD, PTX, sTRAIL + PTX or sTRAIL-iRGD + PTX. (**A**) Cell death was evaluated by TUNEL assay. (**B**) Quantification of tumor necrosis area. (**C**) and TUNEL immunohistochemical positivity. (**D**) Error bars denote SD. *p ≤ 0.05; **p ≤ 0.01; ns, not significant. Scale bar = 100 μm.

Hematoxylin and eosin (H&E)-stained tumor sections showed differences among tumors treated with PBS, sTRAIL, sTRAIL-iRGD, PTX, sTRAIL + PTX or sTRAIL-iRGD + PTX (Fig. 8A,C). Small necrotic regions were present in tumors treated with PBS or sTRAIL Necrotic regions were increased in the sTRAIL-iRGD and sTRAIL-iRGD + PTX-treated groups. Furthermore, tumors from the sTRAIL-iRGD and sTRAIL-iRGD + PTX groups showed stronger TUNEL staining than tumors treated with PBS or sTRAIL, indicating substantial cell death in the sTRAIL-iRGD and sTRAIL-iRGD + PTX-treated tumors (Fig. 8B,D).

## Discussion

Gastric cancer patients exhibit high mortality rates globally^2^, and new low side-effect, high specificity therapeutics are needed for improved treatment outcomes^22^. The use of several available cancer chemotherapy agents is limited due to severe side effects and acquired resistance^23^. Phage display peptide libraries improve the therapeutic efficacy of anticancer drugs by providing peptides with high affinity and specificity. Such peptides typically have low immune reactivity and good tumor and tissue penetration rates^24^. The ability to penetrate deep into a solid tumor is often a great challenge for targeted therapeutics^3^. In solid cancers, homeostatic tissue regulation fails, leading to hypoxia and increased interstitial fluid pressures^25–28^. The extracellular matrix (ECM) can also prevent drugs from moving into tumor tissues^29–31^.

TRAIL, a member of the TNF superfamily, was identified nearly 20 years ago. TRAIL could strongly induce apoptosis in transformed cancer cells and caused no observed negative side effects for the host. TRAIL displays considerable antitumor activity in xenograft models, including colon, breast, multiple myeloma, glioma and prostate cancers^32^. TRAIL induces apoptosis firstly by binding to death receptors (DR4 and DR5), and the death-inducing signaling complex formed upon recruitment of specific cytoplasmic proteins, Fas-associated death domain and caspase-8 or caspase-10^33, 34^. TRAIL is a homotrimeric type II transmembrane protein that can be cleaved proteolytically into a soluble form (sTRAIL) that retains tumoricidal activity. However, recent findings indicate that sTRAIL proteins can negatively affect healthy brain tissue, demic hepatocytes and some epithelial cells, and nonselective sTRAIL gene expression induced liver toxicity^10^.

The iRGD motif is commonly applied for targeting and penetrating tumors^20^, and could be used to enhance sTRAIL tumor specificity, potentially reducing non-target tissue toxicity. In this study, we assessed the therapeutic value of sTRAIL-iRGD as a cancer treatment agent. We showed that sTRAIL-iRGD is effective in suppressing gastric cancer cell line growth *in vitro* and exerts potent tumoricidal activity *in vivo*. The results show that sTRAIL-iRGD increases apoptosis in comparison with sTRAIL in MKN45 cancer cell lines. This increase is attributed to sTRAIL-iRGD selectively binding integrin receptors, increasing its local concentration on target cell surfaces. This permits a more efficient binding of the sTRAIL domain to the proximal TRAIL receptors, enhancing the proapoptotic signal cascade. MTT assay results indicated that sTRAIL-iRGD decreased cancer cell viability. Flow cytometry analysis of cell apoptosis also demonstrated that sTRAIL-iRGD increased MKN45 cell induction of both early and late apoptosis. We believe that the improved sTRAIL-iRGD anti-tumor activity is contributed to the iRGD peptide, which facilitated cell internalization of the recombinant protein. Additionally, sTRAIL-iRGD combined with PTX exhibited enhanced anti-tumor activity as compared with sTRAIL-iRGD alone. Our study showed that sTRAIL-iRGD is an effective antitumor agent that can be disseminated to tumor tissues through intravenous injection.

*In vitro* 3D tumor spheroids serve as helpful intermediates between monolayer cell cultures and animal experiments for assessing drug delivery efficiency. Tumor spheroids produced by liquid overlay techniques contain cell aggregates in close contact and an organized ECM composed of fibronectin, laminin, collagen and GAG, similar to that of tumors *in vivo* ^17^. MKN45 spheroids thus served as a suitable model in our studies to assess the diffusion, penetrance and overall performance of sTRAIL-iRGD. We found that sTRAIL-iRGD penetrated cell lines and tumor tissues more effectively than sTRAIL, indicating that the iRGD peptide facilitated sTRAIL penetration into the 3D tumor spheroids. This efficient transport was also confirmed by growth inhibition experiments, in which sTRAIL-iRGD inhibited MKN45 tumor spheroid growth more so than sTRAIL, although sTRAIL-iRGD in combination with PTX reduce growth most effectively.

Immunohistochemical analyses localized sTRAIL-iRGD within the tumor tissues of xenografted mice at higher levels than sTRAIL. sTRAIL-iRGD treatment effectively inhibited tumor growth *in vivo*, resulting in a nearly 80% reduction in MKN45 cell tumor volume. sTRAIL-iRGD and PTX treatment together reduced tumor volumes more effectively than either therapeutic alone. Mouse lung, kidney, spleen, heart, kidney and other key organs showed no observable toxicity symptom.

In conclusion, this research provides an approach to fusing the sTRAIL protein and iRGD peptide to target tumors. sTRAIL-iRGD had an anti-tumor effect in 2D tumor cell lines, 3D multicellular spheroids and nude mice with tumors. Mice treated with sTRAIL-iRGD and PTX in combination showed no sign of sTRAIL-iRGD–related liver toxicity. Our data suggest that sTRAIL-iRGD is a promising anti-gastric cancer agent with high selectivity and limited systemic toxicity.

## Materials and Methods

### Cell lines and reagents

Fluorescein isothiocyanate (FITC) was obtained from Sigma Chemical Co. (Saint Louis, MO, USA). DAPI was purchased from Beyotime Biotechnology (Wuhan, China). 3-(4,5-Dimethylthiazol-2-yl)-2, 5-diphenyltetrazolium bromide (MTT) was acquired from Sigma Chemical Co. for the purpose of assaying cell viability. The annexin-V-FLUOS Staining Kit was purchased from Roche Applied Science (Indianapolis, IN, USA). Rat monoclonal anti-mouse CD31 and mouse monoclonal anti-human CD31 antibodies were bought from BD Pharmingen (San Jose, CA, USA) and Gene Tech Co. Ltd. (Shanghai, China), respectively. The Cell Death Detection Kit for terminal deoxynucleotidyl transferase–mediated dUTP nick end labeling (TUNEL) was obtained from Roche Applied Science (Indianapolis, IN, USA). The αvβ3 integrin antibody was purchased from Cell Signaling Technology (Danvers, MA, USA). The human NRP-1 antibody was purchased from Abcam (Cambridge, MA, USA). We purchased the human gastric carcinoma cell lines, MKN45 and KATOIII, from the Cell Bank of the Shanghai Institute of Biochemistry and Cell Biology (Shanghai, China).

We cultured all human cell lines in RPMI-1640 (Invitrogen, Carlsbad, CA, USA) with glutamine, streptomycin, penicillin and 10% fetal bovine serum (FBS, Invitrogen) at 37 °C and 5% CO_2_.

### Recombinant protein preparation and characterization

We enrolled a healthy male volunteer for this study. All methods were performed in accordance with the relevant guidelines and regulations by the Institutional Ethics Committees of Drum Tower Hospital, Nanjing University. The human related experimental protocol was approved by the ethics committee of Drum Tower Hospital, Nanjing University. The study was confirmed informed consent by the study participant. The sTRAIL DNA sequence coding for amino acids 114–281 was amplified using reverse transcription polymerase chain reaction (RT-PCR) from volunteer peripheral blood RNA. This study used the following primers: sTRAIL, forward: 5′-CAT GCC ATG GGC *CAT CAC CAT CAC CAT CAC* GTG AGA GAA AGA GGT CCT CAG-3′ (Nco1 site underlined, hexahistidine (His) tag in italics) and reverse: 5′-CCC AAG CTT CTA TTA GCC AAC TAA AAA GGC CC-3′ (HindIII site underlined); sTRAIL-iRGD, forward: 5′-CAT GCC ATG GGC *CAT CAC CAT CAC CAT CAC* GTG AGA GAA AGA GGT CCT CAG-3′ (Nco1 site underlined, hexahistidine (His) tag in italics) and reverse: 5′-CCC AAG CTT CTA GCA GTC CGG ACC TTT GTC ACC ACG GCA *CGA TCC GCC ACC GCC AGA GCC ACC TCC GCC* GCC AAC TAA AAA GGC CC-3′ (HindIII site underlined, iRGD, G4S linker between iRGD and sTRAIL in italics). PCR conditions were as follows: 5 min 94 °C, 30 cycles of 94 °C for 20 s, 58 °C for 30 s, 72 °C for 60 s, with 4 °C extension for 10 min. We digested the PCR products using Nco1 and HindIII, and purified the results using 1% agarose gel electrophoresis with ethidium bromide. The target DNA was ligated into a pET28a (+) *Escherichia coli* expression vector that we had previously cut using Nco1 and HindIII. These enzymes were used to digest the recombinant constructs, which were then sequenced (Invitrogen, Shanghai, China) to verify that the vector construct was cloned with the fusion genes in the correct reading frame. We induced proteins at 37 °C for 4 hours using 0.5 mM isopropyl β-D-1-thiogalactopyranoside and then expressed them in *E. coli* BL21 (DE3). We centrifuged the cells,, then suspended and disrupted them using sonication. We employed nickel-nitrilotriacetic acid affinity chromatography using the AKTA fast protein liquid chromatography system (General Electric Company, USA) to purify the recombinant proteins. We analyzed the eluted fractions using 12% sodium dodecyl sulfate-polyacrylamide gel electrophoresis (SDS-PAGE) and identified them with coomassie brilliant blue. Finally, we conjugated the labeled recombinant proteins with FITC. FITC was dissolved in anhydrous DMSO at 1 mg/ml. For 1 mg protein solution, we added 150 μg FITC solution. The F/P molar ratio was 0.84. The protein concentration was 4.5 mg/ml by BSA detection. We dialyzed and filtered (0.22 mm) the labeled protein.

### Western blotting

Protein extracts were prepared from MKN45 and KATOIII cells (2 × 10^6^) and cultured for 30 min using the following protein lysis buffer: 150 mM NaCl, 1 mM dithiothreitol (Merck, Cefoperazone, Germany), 50 mM Tris–HCl (pH 7.5), 0.1% sodium dodecyl sulfate (SDS) and protease inhibitors (Roche, Basel, Switzerland). Extracts were separated using SDS polyacrylamide gel electrophoresis and electrophoretically transferred to nitrocellulose membrane (Millipore, Waltham, MA, USA). Standard western blotting was conducted using primary antibodies for NRP-1 and DR4.

### Recombinant protein internalization

MKN45 and KATOIII cells in the logarithmic growth phase were seeded at 1.0 × 10^6^ cells/well in 24-well chamber slides (Nalgene Nunc International, USA). Subconfluent tumor cells on the chamber slides were incubated for 1 h with 10 μg/mL FITC-labeled protein. Cells were washed three times with PBS (pH 7.4) and then fixed with ice-cold methanol for 10 min. Cells were again washed three times with PBS (pH 7.4) and nuclei were stained with DAPI. Cells were observed using an LSCM (laser scanning confocal microscope; Zeiss LSM 710).

### Anti-proliferation assay

MKN45 and KATOIII cells in the logarithmic growth phase were seeded at 5 × 10^3^ cells/well in 96-well plates and cultured in a humid environment at 37 °C with 5% CO_2_ for 12 h. Medium was then replaced with serum-free medium containing sTRAIL, sTRAIL-iRGD, PTX, sTRAIL + PTX or sTRAIL-iRGD + PTX. After 24 h incubation, we evaluated cell viability via MTT assay following the manufacturer’s instructions. Cell viability values were calculated using GraphPad Prism v5.0 software (San Diego, CA, USA).

### Flow cytometry

Apoptosis induction was detected using the Annexin-V-FLUOS Staining kit following the manufacturer’s instructions. In brief, cells were seeded at 1.0 × 10^6^ cells/mL at 37 °C with 5% CO_2_. Cells were then treated with 100 ng/ml fusion protein and PTX (4 μg/ml) and cultured for 24 h. Cells were washed twice with cold PBS and treated with 100 μl of Annexin-V-FLUOS for 15 min in the dark at room temperature. Cells were gently vortexed, and flow cytometry analysis was conducted using a FACSort Flow Cytometer (Becton Dickinson, San Jose, CA, USA).

### MKN45 MCS formation and growth inhibition

We used matrigel (BD Bioscience, USA) to generate MCS. The bottom of a 24-well plate was coated with matrigel at 4 °C, and was then incubated at 37 °C for 30 min to assure solidification. MKN45 cells incubated in a monolayer as previously described were trypsinized and collected as a single-cell suspension. 5 × 10^5^ cells in 1 mL of RPMI 1640 medium were seeded into matrigel-treated 24-well plates. Cells were cultured at 37 °C with 5% CO_2_ with daily media replacement. Formation of MKN45 MCS (approximately 200 μm in diameter) occurred spontaneously in seven days. To test drug antitumor effects, we cultured MCS (approximately 200 μm diameter) with serum-free DMEM containing sTRAIL, sTRAIL-iRGD, PTX, sTRAIL + PTX or sTRAIL-iRGD + PTX, with drug-free DMEM as the control. Growth inhibition following drug treatment was evaluated using an ocular micrometer-equipped inverted phase microscope. MKN45 spheroids were measured on days 0, 1, 2, 3, 4, 5, 6 and 7.

### Tumor spheroid penetration

LSCM was used to assess the distribution of sTRAIL-iRGD in MCS. For each experiment, we collected about 20 MKN45 spheroids (approximately 200 μm in diameter) and transferred them to a 5 mL Eppendorf tube. MCS were treated with FITC-labeled sTRAIL-iRGD at various concentrations for 6 or 24 h at 37 °C. Cells were observed using a laser scanning confocal microscope. The ZEN 2008 program(Carl Zeiss MicroImaging GmbH) was used to analyze mean fluorescence intensity in a semi-quantitative mode.

### Animals and tumor tissue penetration *in vivo*

BALB/c mice (male, 18–22 g, 5–6 weeks of age) were bought from BK Lab Animal Ltd. (Shanghai, China) and kept at 25 ± 1 °C under specific pathogen free (SPF) conditions with appropriate food and water. The experimental protocols were approved by the Institutional Animal Care and Use Committee (IACUC) of Nanjing University. Animal handling was conducted according to the protocols of the Institutional Animal Care and Use Committee (IACUC) of Nanjing University.

Five-week old BALB/c nude mice were subcutaneously implanted with 2 × 10^6^ MKN45 cells in 0.1 mL PBS in the right armpits to generate tumor xenografts. FITC-labeled sTRAIL-iRGD or sTRAIL were injected into tumor-bearing mice via tail vein when tumors were approximately 200 mm^3^. One hour after peptide administration, mice were sacrificed and tumors were collected. Immunofluorescence analysis was carried out with frozen tumor tissues. For immunostaining, slides were first blocked at room temperature with 20% goat serum for 1 h, then exposed to an anti-mouse antibody against CD31 (1 × 300 dilution) at 4 °C overnight. Finally, nuclei were stained with DAPI and slides were visualized using an LSCM.

### Tumor treatment in nude mice

Tumor-bearing mice were assigned to six treatment groups approximately two weeks after MKN45 cell inoculation. Group assignment was based on tumor size to ensure that there was no statistical difference in tumor volume amongst the groups when the treatment began. Peptides were diluted in PBS and injected intraperitoneally. A total of four injections were administered into mice every three days. Tumor volume was computed with a digital vernier caliper using the following formula: tumor volume = (length × width^2^)/2, where width was the widest dimension and length was the longest dimension. Experiments were halted when tumor diameter reached 1.5 cm. Mice were sacrificed and xenografts were removed and weighed. We fixed organs, including heart, kidney, liver, spleen and lung, in neutral buffered formalin, and embedded the tissues in paraffin. Sections were stained with hematoxylin-eosin (H&E) for pathological study using optical microscopy. TUNEL assay was used to evaluate tumor cell apoptosis.

### Statistical analysis

All experiments were repeated at least three times. We used Graphpad Prism version 5.0 (GraphPad Software) to analyze data. Data are presented as means ± SD. Data were assessed using the two-tailed Student’s test with Bonferroni correction. Different groups were compared using one-way analysis of variance (ANOVA). The effects of different peptides were compared using two-way ANOVA. P < 0.05 was considered statistically significant.
